# System-level time computation and representation in the suprachiasmatic nucleus revealed by large-scale calcium imaging and machine learning

**DOI:** 10.1038/s41422-024-00956-x

**Published:** 2024-04-11

**Authors:** Zichen Wang, Jing Yu, Muyue Zhai, Zehua Wang, Kaiwen Sheng, Yu Zhu, Tianyu Wang, Mianzhi Liu, Lu Wang, Miao Yan, Jue Zhang, Ying Xu, Xianhua Wang, Lei Ma, Wei Hu, Heping Cheng

**Affiliations:** 1 National Biomedical Imaging Center, State Key Laboratory of Membrane Biology, Institute of Molecular Medicine, Peking-Tsinghua Center for Life Sciences, College of Future Technology, Peking University, Beijing, China; 2 Research Unit of Mitochondria in Brain Diseases, Chinese Academy of Medical Sciences, PKU-Nanjing Institute of Translational Medicine, Nanjing, Jiangsu China; 3 Wangxuan Institute of Computer Technology, Peking University, Beijing, China; 4 Academy for Advanced Interdisciplinary Studies, Peking University, Beijing, China; 5 Beijing Academy of Artificial Intelligence, Beijing, China; 6 College of Engineering, Peking University, Beijing, China; 7 Jiangsu Key Laboratory of Neuropsychiatric Diseases and Cambridge-Su Genomic Resource Center, Medical School of Soochow University, Suzhou, Jiangsu China; 8 Present Address: Department of Bioengineering, Stanford University, Stanford, CA USA

**Keywords:** Calcium signalling, Circadian rhythms, Cellular imaging

## Abstract

The suprachiasmatic nucleus (SCN) is the mammalian central circadian pacemaker with heterogeneous neurons acting in concert while each neuron harbors a self-sustained molecular clockwork. Nevertheless, how system-level SCN signals encode time of the day remains enigmatic. Here we show that population-level Ca^2+^ signals predict hourly time, via a group decision-making mechanism coupled with a spatially modular time feature representation in the SCN. Specifically, we developed a high-speed dual-view two-photon microscope for volumetric Ca^2+^ imaging of up to 9000 GABAergic neurons in adult SCN slices, and leveraged machine learning methods to capture emergent properties from multiscale Ca^2+^ signals as a whole. We achieved hourly time prediction by polling random cohorts of SCN neurons, reaching 99.0% accuracy at a cohort size of 900. Further, we revealed that functional neuron subtypes identified by contrastive learning tend to aggregate separately in the SCN space, giving rise to bilaterally symmetrical ripple-like modular patterns. Individual modules represent distinctive time features, such that a module-specifically learned time predictor can also accurately decode hourly time from random polling of the same module. These findings open a new paradigm in deciphering the design principle of the biological clock at the system level.

## Introduction

The suprachiasmatic nucleus (SCN) is the central pacemaker of the mammalian circadian rhythm. It comprises a pair of oval structures each containing ~10,000 heterogeneous neurons. The central clock integrates the external light and time cues to generate multichannel signals to command peripheral clocks across diverse tissues, thereby regulating physiological functions and daily behaviors of the animal.^1,2^ Almost all SCN neurons are GABAergic along with a plethora of cell type-specific neuropeptides.^3–5^ Individual neurons harbor self-sustained molecular clockwork in the form of the transcriptional/translational feedback loop (TTFL).^6,7^ By regulating the membrane conductance to Na^+^, K^+^ and Ca^2+^, the TTFL signals are further decoded into Ca^2+^ and electrophysiological rhythms, which, in turn, orchestrate the clock gene expression through Ca^2+^/cyclic adenosine monophosphate (cAMP) response elements, and support system-level SCN computation and integration as well as projection to supra-SCN levels.^1^

To date, the exact mechanism underlying time computation and representation by SCN as a whole remains enigmatic. SCN neurons are heterogeneous and exhibit spatiotemporal gradients in terms of clock gene or protein expression, intracellular cAMP and Ca^2+^ signals.^8–13^ The circadian Ca^2+^ rhythm, which originates both endogenously from the core loop and exogenously from the SCN neuronal network, is at least partly dissociable from TTFL, and displays topological specificity, with the phase in the dorsal SCN region ahead of the ventral counterpart in cultured neonatal SCN slices.^14–16^ These observations underscore the importance of intracellular Ca^2+^ in the coupling, integration, and time representation of the SCN network. However, many fundamental questions beg for answers. What is the exact mechanism whereby these spatiotemporal gradients and topological specificities are decoded into the time of day? Given that a small subset of SCN neurons is critical for maintaining rhythmic function,^17^ do SCN neurons contribute uniformly, or else some subpopulations dominate in this process? How many neurons are required to make a robust time signal, in order for the master clock to generate multi-timed outputs to inform diverse peripheral clocks?

At present, deciphering the design principles of complex nuclei such as SCN is still a formidable task, and there is an urgent need for innovative technology and methodology to provide new tools and insights. In this study, we applied high-speed dual-view two-photon microscopy for volumetric Ca^2+^ imaging of 6000–9000 GABAergic neurons in adult SCN slices, and captured multiscale Ca^2+^ signals ranging from seconds to minutes to hours and up to an intact circadian period. Leveraging the power of machine learning methods, we revealed a modular organization of time feature representation that gives rise to bilaterally symmetrical ripple-like patterns in the SCN space. Importantly, we unveiled a time-prediction mechanism based on polling just a few hundred neurons of the SCN, with all of them being equally important in this process.

## Results

### Multiscale Ca^2+^ signals in the SCN

**Fig. 1:**
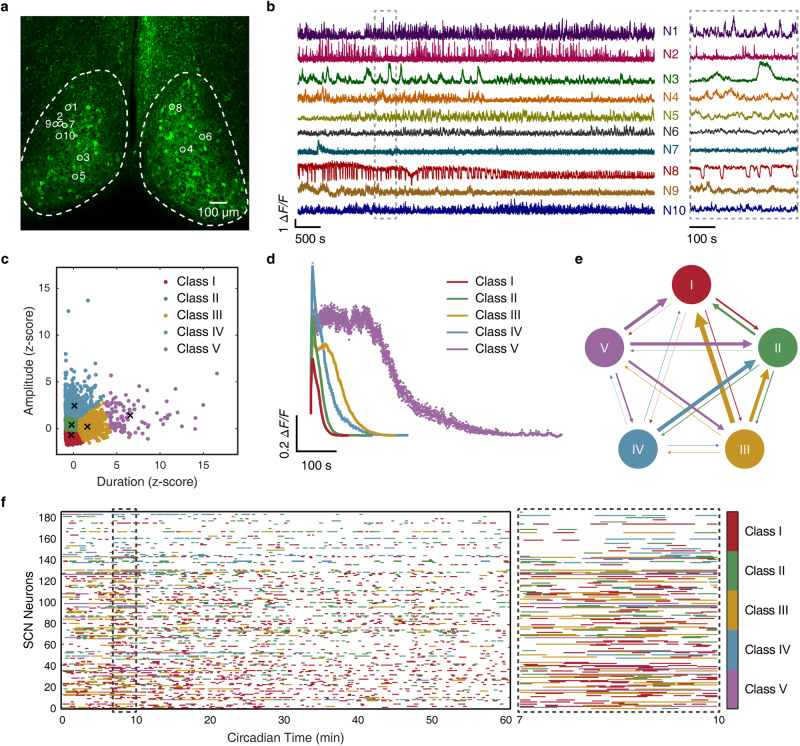
Multiscale Ca^2+^ activities in SCN GABAergic neurons. **a** A representative two-photon image showing GABAergic neurons expressing GCaMP6f in an SCN slice from an 8-week-old *Viaat-Cre::GCaMP6f* mouse. Dashed lines demarcate the estimated borders of the SCN. Scale bar, 100 μm. **b** Diversity of SCN neuronal Ca^2+^ signals. Data shown are 1-h excerpts from a total of 6-h continuous recording at a 2.7 Hz frame rate, and corresponding neurons are marked in **a**. Right, an enlarged view of the segment in the dashed box. **c** Clustering of Ca^2+^ bursts based on their duration and amplitude by a K-means clustering algorithm. Crosshairs mark the centroid of 5 classes identified. **d** Time courses of averaged Ca^2+^ bursts in different classes. Data are shown as mean ± SEM (*n* = 4643, 3150, 764, 638, and 100 events for class I, II, III, IV, and V, respectively). **e** Schematic diagram of inter-class switching of Ca^2+^ bursts. The direction and relative thickness of the arrow represent the direction and relative propensity of switching, respectively. **f** Raster plot showing the kinetics of inter-class switching of Ca^2+^ bursts. Right, an enlarged view of the segment in the dashed box.

We applied two-photon microscopy to image freshly isolated SCN slices from adult *Viaat-Cre::GCaMP6f* mice with their GABAergic neurons expressing the genetically coded Ca^2+^ indicator GCaMP6f. To capture temporal properties of different scales, time-lapse images were acquired at 2.7 Hz for 6 h without any interruption (Fig. 1a). In all GCaMP6f-expressing neurons, transient, discrete Ca^2+^ events, namely Ca^2+^ bursts, occurred atop slow baseline Ca^2+^ oscillations (Fig. 1b). Intriguingly, SCN Ca^2+^ bursts varied drastically by virtue of frequency, amplitude, duration and, in rare cases, polarity; their waveforms were polymorphic, being spiky, or triangle-shaped with a slow descending slope, or echelon- or square-shaped with a conspicuous plateau (Fig. 1b). When Ca^2+^ bursts were categorized by a K-means clustering algorithm based on duration and amplitude, we identified five classes of Ca^2+^ bursts as determined by the gap statistic method (Supplementary information, Fig. S1a),^18^ with average durations spanning from 17.4 s to 129 s at 5% and 95% percentiles (Fig. 1c, d; Supplementary information, Fig. S1b, c). The frequency of Ca^2+^ bursts in the SCN neuronal population displayed a broad bell-shaped distribution, ranging from 0.001 Hz to 0.011 Hz at 5% and 95% percentiles; and the inter-Ca^2+^ burst intervals followed a roughly exponential distribution with a long tail (Supplementary information, Fig. S1d, e). Notably, the vast majority of neurons underwent frequent switching between different classes of Ca^2+^ bursts, with a tendency being switched to classes of shorter duration (Fig. 1e, f; Supplementary information, Fig. S1f). This result suggests that Ca^2+^ bursts of distinctive classes likely constitute fundamental units to form higher-order temporal features for time representation and computation in the SCN.

Encouraged by these observations, we sought to investigate population-level Ca^2+^ activity in the entire SCN over an intact circadian period. However, we met with an unforeseen challenge that two-photon microscopy can penetrate SCN only to the depth of ~200 μm at a tolerable laser intensity. To overcome this limit, we designed and custom-built a dual-view two-photon microscope, both inverted and upright, doubling the penetration depth and fluorescence collection efficiency while lowering photodamage. The perfusion system was also adapted for temperature control, sterilization, and dual-side perfusion of the slice over a prolonged culture time (Supplementary information, Fig. S2). Meanwhile, to improve the signal-to-noise ratio, we opted to use *Viaat-Cre::GCaMP6s* mice for brighter fluorescence signals.^19^ Using this system, we performed time-lapse volumetric imaging of 650 μm × 650 μm × 300 μm SCN slices at 0.67 volumes/s for 5-min recording every hour up to 30 h, and obtained six complete datasets (Supplementary information, Table S1). In a typical volume stack, we extracted Ca^2+^ time series from 6000–9000 neurons along with their spatial coordinates, via a custom-devised image analysis pipeline (Supplementary information, Fig. S3).

**Fig. 2:**
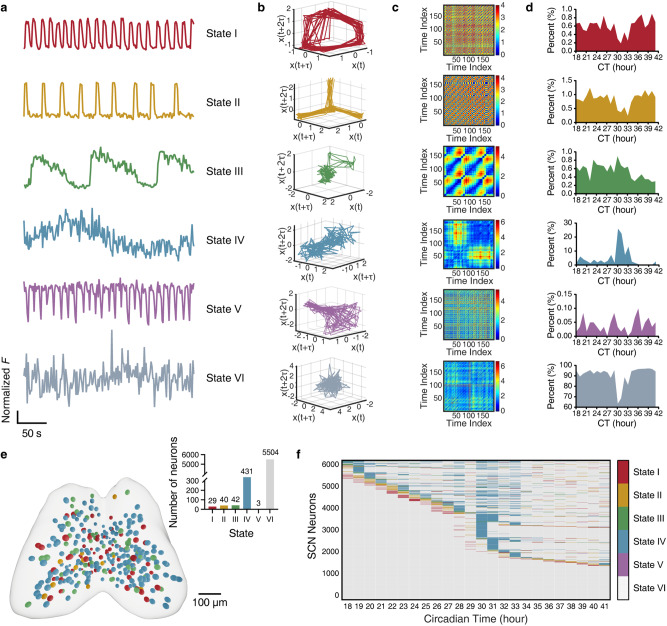
Ca^2+^ states and state-switching dynamics in SCN neurons. Data were from a slice containing 6049 identified neurons. **a** Representative 5-min Ca^2+^ recordings, corresponding to different Ca^2+^ states of SCN neurons (State I to State VI). Data shown are *Z*-score normalized fluorescence intensity. **b**, **c** Phase-space manifolds (**b**) and recurrent plots (**c**) of corresponding traces in **a**. Note the repeating features or motifs of systematic dynamics in the recurrent plot. **d** Percentage of neurons at different Ca^2+^ states over a 24-h timescale. See Supplementary information, Fig. S4 for neuronal Ca^2+^ state classification. **e** Spatial distribution of neuronal Ca^2+^ states at CT28. The most populous State VI neurons (inset, top right) are omitted for clarity. **f** Raster plot of state-switching over 24 h for all neurons. The color codes of Ca^2+^ states are shown on the right.

As judged from the kinetics of inter-class switching of Ca^2+^ bursts, we assumed that an SCN neuron stays at the same functional state within a short time period (e.g., 5-min). It follows that the ensemble of all 5-min recordings collected from thousands of neurons over a circadian period shall cover virtually all Ca^2+^ states attainable by SCN GABAergic neurons. To this end, we resorted to machine learning technology to develop an automated Ca^2+^ state classifier. We first transformed Ca^2+^ time series into phase-space manifolds,^20–22^ and summarized them into six states contingent on visual observation (Fig. 2a–c). Subsequently, we constructed and trained a graph convolutional network (GCN)^23–25^ to assign Ca^2+^ states to individual neurons based on their 5-min Ca^2+^ behavior (Supplementary information, Fig. S4). The simplest manifold came from State I (0.60% in total), showing a ring-shaped structure, while the most complex manifold came from State II (0.84% in total), showing a three-pointed star. The predominated state, State VI (93%), exhibited fast, irregular, and nonperiodic fluctuations in the time domain, and gave rise to a stochastic oscillating manifold in the phase space, whereas State V was the rarest (0.04%, Fig. 2d) and characterized by brief downward deflections from consistently elevated Ca^2+^ levels. When mapped to the SCN space, all these minority states entwined irregularly and the mosaic pattern variably evolved at different circadian times (Fig. 2e; Supplementary information, Fig. S5).

Over a 24-h period, while waxing and waning through different Ca^2+^ burst classes, most neurons also displayed robust inter-state switching behavior (Fig. 2f), hinting at the existence of even higher-order temporal features of Ca^2+^ signals. Indeed, when Ca^2+^ signal amplitude (mean Δ*F/F* over a 5-min recording) was analyzed as a function of time, we revealed significant differences between two 12-h divisions, i.e., high-activity Ca^2+^ mode (H-mode) and low-activity Ca^2+^ mode (L-mode) over a 24-h period. Such circadian Ca^2+^ rhythmicity was confirmed in almost all SCN neurons examined, along with neuron-to-neuron variability in the timing of inter-mode transition (Supplementary information, Fig. S6).

Notably, a dramatic event switching to State IV occurred around circadian time (CT) 30 (Fig. 2f). By reviewing this event in the SCN space, we identified an SCN-wide activity, namely phase wave of hyperactivity (PWHA), which originated from the peripheral SCN, entered the SCN at the dorsal tip (CT26), then propagated to the ventromedial region (CT28–CT29), triggered a global Ca^2+^ excitation (CT30) before fading out (CT31–CT38) (Supplementary information, Fig. S7 and Video S1). This finding is consistent with previous reports on extremely slow Ca^2+^ waves in cultured neonatal SCN.^10,16^ The observation that PWHA lasted on a 12-h timescale and swept across the entire nucleus indicates that it represents a spatiotemporal feature of the largest scales in the SCN.

Taken together, we uncovered a full spectrum of multiscale Ca^2+^ events at the SCN neuronal and ensemble levels, ranging from elemental Ca^2+^ bursts (seconds to minutes) to Ca^2+^ states (minutes to hours) to Ca^2+^ modes as well as population-level PWHA (~12 h). This finding underscores the idea that time-keeping by SCN is much more sophisticated than simply maintaining global coherent oscillations via synchronization.

### Ensemble Ca^2+^ signals predict hourly time

To investigate whether and how multiscale SCN Ca^2+^ signals encode and decode time information, we quantified neuronal and ensemble Ca^2+^ behaviors in relation to physical time. We reckoned that, as Ca^2+^ signals are intimately related to SCN inputs and outputs, understanding the time-keeping principles of the SCN can be naturally cast as learning the feature representation of the multiscale Ca^2+^ activity.

**Fig. 3:**
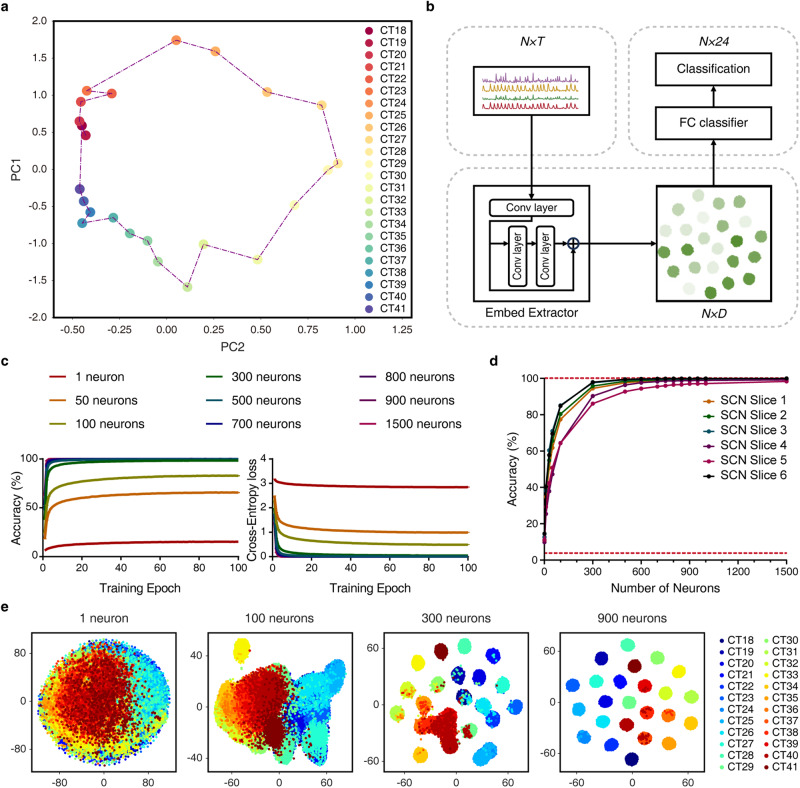
Hourly time prediction by polling randomly selected cohorts of SCN neurons. **a** Circular evolution trajectory of a 2D representation of population-level Ca^2+^ activities. Axes correspond to the first two principal components, PC1 and PC2. Different time points are represented in different colors, and consecutive time points are connected by dotted lines. **b** Scheme of time-predictor based on SCN Ca^2+^ signals. Conv and FC refer to the convolutional and fully connected layer; *N*, *T* and *D* denote the dimensions of neurons, the input Ca^2+^ time series of each neuron and its features extracted, respectively. **c** Accuracy and loss curves during training process. **d** Prediction accuracy curves, showing results from six SCN slices. Data are shown as mean ± SEM (*n* = 5000 trials). Lower dashed line represents the chance level. **e** Visualization of CNN’s high-dimensional features in a 2D space via t-SNE. From left to right: the number of neurons is 1, 100, 300, and 900, respectively.

Taking all observed neurons in each SCN as a whole, we employed principal component analysis^26–28^ to appraise the collective properties of all the 5-min Ca^2+^ time series in a low-dimensional space. We found that the resultant data points in the PC1–PC2 space delineated a circular temporal evolution trajectory (Fig. 3a). This result inspired us to further explore whether one could utilize SCN Ca^2+^ signals to predict physical time and, in so doing, highlight the SCN’s core function as a time-keeping system.

We therefore employed modern deep learning tools^29,30^ to decode time information from SCN Ca^2+^ signals. Using a convolutional neural network (CNN) consisting of one convolutional layer, a residual block followed by one fully connected (FC) layer, we achieved hourly time prediction by involving randomly selected cohorts of neurons (Fig. 3b, c). To be specific, single-neuron Ca^2+^ signals could predict the correct time at a slightly above-the-chance level, supporting their ability of time feature representation. Prediction accuracy steeply increased with the size of the cohorts polled and reached 99.0% ± 0.4% (mean ± SEM, *n* = 6) at a cohort size of 900. Even higher accuracy of time prediction could be attained by polling a still greater number of neurons (Fig. 3d). To confirm that such discrimination of time truly reflects disparities hidden in the Ca^2+^ time series, we visualized the output features of the second convolutional layer in a two-dimensional (2D) space via t-distributed stochastic neighbor embedding (t-SNE).^31^ Clearly, as the cohort size increased, the data points gradually congregated into distinctive clusters in the feature space, and became more compact within clusters and more separable between clusters (Fig. 3e). Thus, hourly time prediction by SCN is contingent on the integration and computation of Ca^2+^ signals from cohorts of neurons, analogous to group decision-making in a statistical system.

Next, we estimated the contribution coefficient for each and every neuron from the integrated gradient-based attribution map after min-max normalization.^32^ Histograms of contribution coefficients of individual neurons were well fitted to a Gaussian distribution, at all time points examined (Supplementary information, Fig. S8a), while for a given neuron, its contribution coefficient fluctuated over time (Supplementary information, Fig. S8b). However, after being averaged over an intact circadian period, single-neuron contribution coefficient exhibited a very narrow Gaussian distribution, with a mere 7% difference between the 5% and 95% percentiles (Supplementary information, Fig. S8c). Thus, our quantitative assessment suggests that, on a 24-h scale, all SCN neurons contribute uniformly in terms of time computation.

### Modular time representation in the SCN

**Fig. 4:**
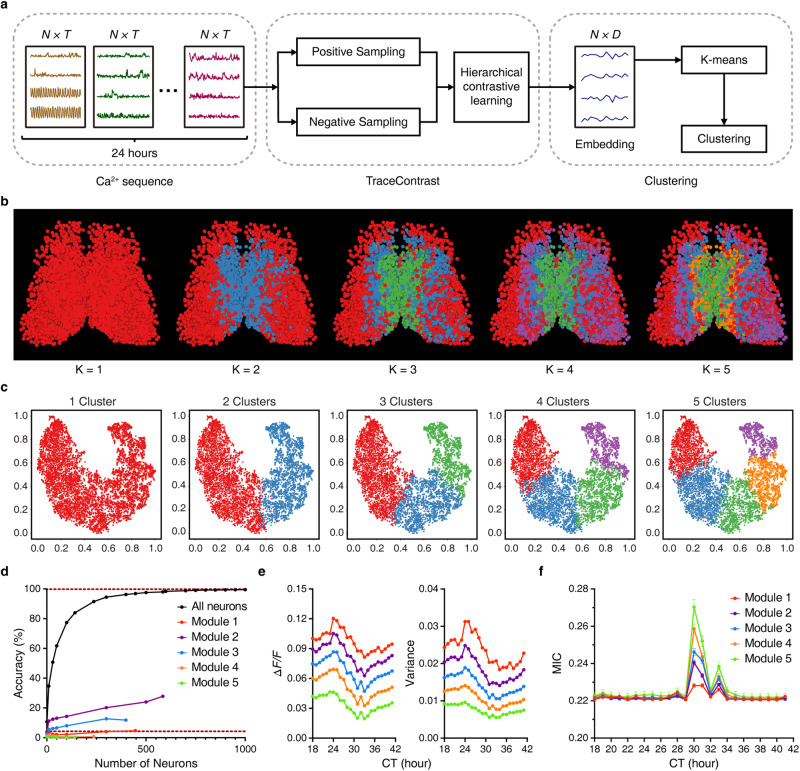
Modular time feature representation revealed by TraceContrast. **a** Scheme of neuron subtype classification by TraceContrast. *N*, *T*, and *D* denote the dimensions of neurons, the input Ca^2+^ sequence of each neuron and its output features, respectively. **b** Bilaterally symmetric and hierarchical modularity emerged from neuron subtype classification. The predefined number of clusters (K) is listed below each corresponding image. **c** The t-SNE plots of the dimensionality reduction corresponding to **b**. **d** Time predictability when sampling only within one specific module (K = 5). Lower dashed line represents the chance level. Data are shown as mean ± SEM (*n* = 5000 trials). Note that one-module-only sampling results in a marked disruption to time prediction accuracy compared to random sampling in all SCN neurons. Similar results were obtained when K = 2, 3, 4. **e**, **f** Ca^2+^ signal amplitude and corresponding variance (**e**) and average MIC in different modules (**f**). Data are shown as mean ± SEM (*n* = 1587, 1936, 1211, 835, and 480 for Modules 1, 2, 3, 4 and 5, respectively). Note that the two attributes showed smooth inter-modular gradients running in opposite directions.

Classic topological division of the SCN is based on retinal and efferent connectivity and expression of neuropeptides, with VIP/GRP in the ventral “core” region and AVP in the dorsal “shell” region.^33,34^ Regional oscillators in the SCN, namely the evening oscillator and the morning oscillator, and the light-responsive area exhibit photoperiodic changes in response to different external photoperiods.^35,36^ Recently, single-cell RNA-sequencing has allowed for fine-grained cell type classification, identifying five SCN neuron subtypes occupying distinct spatial domains.^37^ To identify functional organization patterns hidden in the ensemble Ca^2+^ behavior of SCN, we cast the problem of functional neuron subtype classification as time-series representation learning in an unsupervised manner, and built a classifier TraceContrast (Fig. 4a). In this classifier, we leveraged contrastive learning,^38^ a cutting-edge unsupervised machine learning paradigm, in which Ca^2+^ time series augmented with transformations such as masking and cropping (i.e., positive samples) were contrasted against those from different timestamps or neurons (i.e., negative samples) in a hierarchical fashion. By enforcing the consistency among features of positive samples, TraceContrast should be able to capture emergent properties of SCN Ca^2+^ signals that are invariant to transformations.

By TraceContrast, we categorized all observed neurons from the same SCN slice into 2, 3, 4, or 5 functional subtypes (Fig. 4; Supplementary information, Fig. S9). Using t-SNE to visualize the learned high-level features, we found that the clusters were well separated after their dimensionality reduction into 2D space (Fig. 4c). Spatial mapping revealed that same-type neurons aggregated in the SCN space, giving rise to distinctive ripple-like patterns with bilateral symmetry (Fig. 4b; Supplementary information, Video S2). With an increasing number of subtypes, fine-grained modules were peeled off layer by layer in a coherent and continuous manner, indicating a modular and hierarchal time feature representation by Ca^2+^ signals in the SCN.

We tested the robustness of modular organization in the SCN. We showed that PWHA appeared to exert no effects on the modular organization for two reasons. Firstly, the diffusion direction of the modular ripple did not align with that of PWHA. Secondly, by division of the 24-h Ca^2+^ sequence into three 8-h segments, with no PWHA in the first segment, the modular ripple remained intact for all three subsets of data, affirming that it is indeed PWHA-independent (Supplementary information, Fig. S10a–c). By spatial and temporal subsampling, we showed that, albeit noisier, the bulk of the modular ripple was unchanged when only 50% of neurons were retained by the farthest point sampling method^39^ (Supplementary information, Fig. S10d) or when every other data point in 5-min recordings were dropped out (Supplementary information, Fig. S10e). Furthermore, symmetric modular ripple emerged even from single-sided analysis using either left or right SCN data independently. Altogether, we conclude that the modular ripple-like organization is an emergent property pertaining to time feature representation by the SCN at work.

**Fig. 5:**
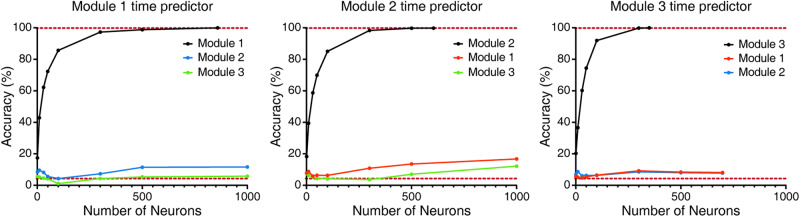
Module-specific time predictors and their performance. Dataset was the same as in Fig. 4 with K = 3. Three module-specific time predictors were trained, each using data from the pertinent module, and its performance pertaining to hourly time prediction was tested in all 3 modules, in order to assess their same-module and cross-module time predictability. The lower dashed line in each panel represents the chance level. Data are shown as mean ± SEM (*n* = 5000 trials).

Using the time predictor described above, we demonstrated that single-module sampling resulted in a markedly degraded performance as compared with random sampling across all modules (Fig. 4d). To further explore topological specificity, we generated module-specific time predictors by training each of them with data from one module only. We showed that the accuracy attainable for same-module time prediction was still greater than 99%, but its cross-module time prediction ability was almost completely abolished (Fig. 5), suggesting that each module has a full yet unique representation of time features.

To gain insight into the nature of invariant properties captured in contrastive learning, we analyzed module-specific Ca^2+^ signal amplitude and variance, and uncovered their ordered variation over modules, manifesting as a smooth ventrolateral-to-dorsomedial upward gradient (Fig. 4e). We also measured the maximal information coefficient (MIC),^40,41^ an index of the coupling strength within a module. While changing dynamically over time, MIC also exhibited an ordered spatial gradient, except that it ran in the opposite direction (Fig. 4f). In other words, our neuron subtype classification might have captured Ca^2+^ signal attributes and coupling strength as invariant properties.

## Discussion

In dissecting system-level mechanisms underlying SCN rhythmicity, we performed volumetric Ca^2+^ imaging of 6000–9000 GABAergic neurons in each adult SCN slice using a high-speed dual-view two-photon microscope, and devised hierarchical sampling strategies to retain multiscale features inherent to SCN Ca^2+^ signals. Furthermore, we introduced cutting-edge machine learning methods to extract hidden features and emergent properties from massive datasets so acquired, with an emphasis on network-level time feature representation and topological organization.

We have uncovered a number of salient and rather distinctive features of SCN Ca^2+^ signals. First, unlike Ca^2+^ signals in other brain regions,^42^ the most striking fact is that Ca^2+^ bursts in the SCN are highly diverse in amplitude, duration, waveform, and even polarity, and inter-Ca^2+^ burst intervals vary across two orders of magnitude. Second, Ca^2+^ bursts of five classes constitute the elemental units to build higher-order temporal features, including six Ca^2+^ states of minute-to-hour duration and two Ca^2+^ modes switching over a circadian period. The Ca^2+^ burst class-, Ca^2+^ state- and Ca^2+^ mode-switching occur spontaneously and incessantly, and its choreography in space may underlie the SCN-traversing, 12-h PWHA similar to Ca^2+^ waves reported previously.^16^ These multiscale spatiotemporal features of Ca^2+^ dynamics would provide a rich, system-level toolkit for the mammalian central clock to base its time feature representation and computation. Our observation supports the notion that the heterogeneity of SCN neurons and spatiotemporal gradients play a crucial role in encoding time-of-day through collective processes.^8–13,35,43–45^

Several lines of evidence indicate that SCN Ca^2+^ signals are indeed time-computing and representing via a group decision-making mechanism. Hourly population-level Ca^2+^ activities delineate a 2D circular evolution trajectory after dimensionality reduction. Leveraging the power of machine learning methods, we successfully created a time predictor with 99% accuracy of time prediction on an hourly scale by polling merely hundreds of randomly selected neurons. As it takes ~900 neurons to attain 99% accuracy, at least twenty or so output channels of hourly precision can be sorted out from the central clock harboring ~20,000 neurons to command diverse peripheral clocks. With non-interruptive whole-nucleus Ca^2+^ recording in the future, one could explore whether SCN population-level Ca^2+^ signals could predict time on finer biologically relevant scales, and how this time-keeping mechanism might be perturbed by pathological and experimental interventions.

Pertinent to this observation, it is important to note that, after time-averaging, the single-neuron contribution coefficient to time prediction obeys a very narrow Gaussian distribution. To the first approximation, this finding suggests that all SCN neurons are strikingly homogenous in terms of contribution to time prediction. Given the hour-to-hour fluctuations of single neurons’ contribution coefficients, a dynamic load-balancing scheme might be at work to shift and equalize the workload among individual neurons. In this scenario, frequent state- and mode-switching as well as the spatiotemporally ordered PWHA might contribute to workload equalization. Some neuropeptide-mediated interplays among closely packed neurons might also be at work, and these possibilities merit future investigations.

A novel type of topological specificity emerged as we applied contrastive learning technology to classify functional neuron subtypes. Neurons of the same functional subtypes aggregate physically and different subtypes are organized in ripple-like modules, aligning with the opposing gradients of Ca^2+^ signal amplitude and coupling strength. Together with the finding on spatiotemporal clusters of PER2::LUC identified by a K-means clustering,^13^ the present and previous findings reinforce the notion that spatiotemporal gradients and topological specificities serve as conserved features of SCN that penetrate different physiological levels ranging from genes and proteins to Ca^2+^ activities, while being manifested across multiple spatial and time scales. Importantly, we showed that each module has unique features gauged by Ca^2+^ signal amplitude and variance as well as MIC, and such module-specific Ca^2+^ signals allow for the module-specifically trained time predictors to accurately decode time of the day based on single-module Ca^2+^ signals. Teleologically, such a modular organization might endow SCN outputs with diversity as well as tuning ability through combinatorial polling and sorting. This point-of-view is in line with the speculation that region-specific SCN projection to downstream brain areas may cater to distinctive demands of different peripheral clocks.^46^

Overall, our findings support a statistical working model for SCN as the mammalian circadian time-keeping system. Multiscale spatiotemporal Ca^2+^ dynamics at neuronal and ensemble levels constitute the building blocks of time representation and computation. Individual neuron each carries only piecemeal time information, and different functional subtypes of neurons represent distinctive time features. Accurate time-keeping can thus be achieved via a group decision-making mechanism, i.e., polling sufficiently large cohorts of neurons. The finding that neurons switch between states and modes as well as the presence of PWHA might reflect a dynamic load-balancing scheme to equalize contribution coefficients from individual members in the system. Finally, topological specificity, as evidenced by the modular organization of time features, may endow multi-timed output signals with diversity and tuning ability, such that one master clock can command and coordinate a variety of peripheral clocks.

## Materials and methods

### Animals

All animal experiments were conducted in accordance with the guidelines of the Animal Care and Use Committee of Peking University accredited by AAALAC International and the procedures were approved by the Animal Care Committee of PKU-Nanjing Institute of Translational Medicine (Approval ID: IACUC-2021-023). All mice were housed in a temperature-controlled (20–22 °C) and specific pathogen-free animal facility, 3–5 per cage and maintained on a 12-h light/dark cycle, with water and food available ad libitum. The experimental mice expressing GCaMP6s or GCaMP6f specifically in GABAergic neurons (*Viaat-Cre::GCaMP6s* mice or *Viaat-Cre::GCaMP6f* mice) were generated by crossing *Viaat-Cre* mice (JAX #017535)^47^ with *Rosa26-LSL-GCaMP6s* mice^48^ or *Rosa26-LSL-GCaMP6f* mice (JAX #024105)^49^ for at least two generations.

### Preparation of SCN slices

Before the experiment, male mice of 4–6 weeks old were individually housed in cages containing running wheels for 14 days, with light on from 07:00 to 19:00 in a 12-h light/dark cycle. Their locomotor activity was recorded and analyzed using ClockLab (Actimetrics). The mice exhibiting robust rhythmic locomotor activity were chosen for the subsequent preparation of SCN slices.

The mice were removed from the home cage at zeitgeber time 11, anesthetized with 1.25% tribromoethanol (20 μL/g, i.p.; EasyCheck), and subsequently transcardially perfused with ice-cold section artificial cerebrospinal fluid (aCSF) containing 110 mM choline chloride, 25 mM NaHCO_3_, 25 mM d-glucose, 7 mM MgCl_2_·6H_2_O, 2.5 mM KCl, 1.3 mM NaH_2_PO_4_, 0.5 mM CaCl_2_, 1.3 mM l-sodium ascorbate and 0.6 mM sodium pyruvate saturated with 95% O_2_/5% CO_2_ at pH 7.3–7.4.^50,51^ Then the mice were decapitated followed by brain removal. The optic nerves were carefully cut to ensure that no stretching occurs to damage the SCN. The brain was then rapidly immersed in ice-cold section aCSF and sliced using a vibratome (VT1200, Leica). A 300-μm coronal slice containing the middle-rostrocaudal region of the SCN was prepared and incubated in recording aCSF (124 mM NaCl, 24 mM NaHCO_3_, 10 mM d-glucose, 3 mM KCl, 1.25 mM NaH_2_PO_4_, 2 mM CaCl_2_ and 1 mM MgSO_4_) at 34 °C for 30 min.^52^ The recording aCSF was bubbled with 95% O_2_/5% CO_2_ for 1 h before use. After incubation, the SCN slice was transferred to a submerged slice chamber (RC-27LD, Warner Instrument) and then placed on a heating platform (PM-7D, Warner Instrument) for subsequent Ca^2+^ imaging. To allow continuous perfusion of the SCN slice from both the upper and lower sides, a custom-made slice supporter (threads are made of Lycra and spacing 1 mm) and a slice anchor (2 mm thread spacing, SHD-27LP/2, Warner Instrument) were used to support it 0.5 mm above a cover glass (24 mm × 60 mm, #1 thickness, Deckglaser). Persistent perfusion of oxygenated recording aCSF heated to 34 °C was conducted by a peristaltic pump (BT100-2J, LongerPump) and a vacuum pump (7E-C, Yuwell) at a rate of ~3 mL/min.

### Ca^2+^ imaging of SCN slices

A two-photon microscope equipped with a Ti:Sapphire laser (Chameleon Vision S, Coherent) and a 25× objective (Apochromat, NA1.10, Nikon) was used to continuously image Ca^2+^ signals reported by GCaMP6f. Images were acquired with two-photon excitation at 920 nm and emission collection at 500–550 nm. The time-lapse images (1024 × 960 pixels, 0.65 μm pixel size) were obtained at 2.7 Hz for 6 h without any interruption.

For volumetric Ca^2+^ imaging of the whole 300-μm SCN slice, we designed and custom-built a resonant scanning dual-view two-photon microscope to acquire time-lapse images. The setup involved two resonant scanning two-photon microscopes positioned symmetrically on the upper and lower sides. Both microscopes were equipped with identical objectives (25×, Apochromat, NA1.10, Nikon) and utilized the same design of excitation and collection light paths. The upright microscope captured images of the upper half of the slice, while the inverted microscope captured images of the lower half. Images were acquired alternately from the upper and lower sides. The light source was a Ti:Sapphire laser (Chameleon Vision S, Coherent) running at 920 nm. The GCaMP6s fluorescence was reflected by a dichroic mirror (FF705-Di01, Semrock) through a bandpass filter (FF03-525/50, Semrock). The laser power at different imaging depths was pre-calibrated and adjusted accordingly. To enhance the signal-to-noise ratio and optimize collection efficiency, the fluorescence signals from either side of the microscope were simultaneously detected and then combined.

*Z*-axis scanning was performed with both objectives using a piezo objective scanner (PFM450E, Thorlabs). Each volumetric stack comprised 44–48 layers (512 × 512 pixels per layer, 1.27 μm pixel size) with intervals of ~6.7 μm, covering a ~300 μm-thick SCN slice. Continuous volumetric imaging was conducted at a rate of ~0.67 volumes/s for 5 min. The imaging protocol commenced at CT12 and was repeated hourly for 30 h. The last 24-h imaging data were used for analysis, allowing time for equilibration and device stabilization.

### 3D-t image analysis pipeline

Images were processed using a custom-developed 3D-t image analysis pipeline based on MATLAB (Mathworks) and Python platform. First, to align and combine the images of the upper and lower sides into a coherent whole-SCN t-series stack, we used piecewise rigid motion correction NoRMCorre to correct slight displacements between the two sides.^53^ Next, non-rigid motion correction NoRMCorre was applied within each time point to generate average-projection image stacks. Based on the average-projection image stacks, we employed the elastix algorithm for 3D image registration between each time point.^54^ Then, we employed a correlation-based method to generate feature images that amplify the contrast between Ca^2+^ activity and background noise. This method operates on the principle that pixels associated with a neuron typically exhibit synchronized temporal dynamics, thus showing a higher correlation.^55,56^ Subsequently, we created hybrid images by fusing these feature images with average-projection images, thereby integrating both morphological and activity-based information. Neuronal regions were identified using the UNet2DS algorithm applied specifically to these hybrid images.^57^ The segmented regions of interest (ROIs) were then manually proofread. To define SCN neurons, we segmented them within an empirically delineated SCN outer contour. This contour served as a boundary to differentiate between SCN and extra-SCN regions.

Subsequently, neuronal Ca^2+^ time series were extracted. The fluorescence intensity (*F*) of a certain neuron was calculated as the averaged fluorescence after background subtraction from the raw image fluorescence (*F*_*raw*_), i.e., *F* = < *F*_raw_ – *F*_bg_ >, where brackets denote the average over the pixels within the ROI region, *F*_*bg*_ denotes the background baseline fluorescence which was calculated as the mean of the lowest 10% fluorescence pixels of the image. For a given fluorescence signal, the MLspike algorithm was employed to extract Ca^2+^ burst attributes,^58^ such as Ca^2+^ burst onset time and baseline fluorescence intensity (*F*_*0*_). The Ca^2+^ burst amplitude was measured as the peak Δ*F/F*, i.e., (*F* – *F*_0_)/*F*_0_ of the respective Ca^2+^ burst event. The duration of individual Ca^2+^ burst was measured as the full width at half maximum (Supplementary information, Fig. S3e).

### Graph-based Ca^2+^ state classification

The fluorescence signals were first subjected to *Z*-score normalization. Following normalization, these signals were projected onto a phase-space manifold that encompasses all potential states of the system. During this transformation, we utilized mutual information to optimize the interval between consecutive data points in the manifold — termed “delay time”, effectively reducing redundancy.^59^ Next, we specified the requisite number of coordinates for representing the system’s dynamic states within the phase-space to be three, referred to as “embedding dimension”. This 3D construction was subsequently organized into a directed graph, where data points were nodes linked by directed edges. This directed graph representation allowed us to cast the classification of “Ca^2+^ states” for all neurons (Fig. 2) as a graph classification problem.

The correspondence between each of the six Ca^2+^ states identified empirically and its phase-space topological manifold is shown in Fig. 2, and the phase-space topological manifolds for 429 neurons were manually labeled. Further, data augmentation was performed to acquire more training data to expand the training set by a factor of four, by (1) perturbing the magnitude by multiplying a factor with mean 1 and variance 0.5; (2) swapping the first and second halves of each state. The final training dataset comprised a total of 832 instances. Among them, 25% were original training data, while the remaining 75% were augmented data. Then, a classical graph classification method known as the GCN^23^ was adopted to classify phase-space topological manifolds into one of the six predefined states. The GCN architecture consisted of three layers of graph convolution kernels, with the dimension of the intermediate layer set to 32. We implemented a Leaky ReLU activation function, applying a negative slope of 0.2. We utilized the Adam optimizer (learning rate, 0.001; training duration, 160 epochs). The outputs from the three layers were then concatenated together and processed through global max pooling to acquire graph-level features. The resulting pooled graph-level representation was fed into a classifier composed of two FC layers to obtain the final classification results. The classifier was optimized by Cross-Entropy loss. Finally, the trained model was adopted to categorize Ca^2+^ states for every 5-min time series.

### Visualizing spatiotemporal PWHA

To generate contour plots of PWHA in selected SCN layers, we first employed spatial smoothing of the average-projection image using MATLAB’s built-in function *imgaussfilt* with a sigma of 3.5. This is followed by intensity normalization for each layer, dividing by its mean pixel value. Pixels with a second derivative exceeding a set threshold are marked as “excited”.^60^ These excited pixels at synchronized timings outline the PWHA wavefront, as shown in Supplementary information, Fig. S7.

To provide a 4D (*XYZ*-t) view of PWHA, we developed a custom visualization software using the Unity platform. Individual neurons were represented as particles using Unity’s VFX Graph system (https://unity.com/visual-effect-graph). The particle system is a technical concept in computer graphics used to visualize a range of complex and high-dimensional phenomena. The rendering of each particle encodes a neuron’s 3D spatial coordinate, normalized fluorescence signals (brightness), and subtype (color). To reveal spatiotemporal patterns, normalized fluorescence signals were temporally averaged over 200 frames (5-min recording). A sampling curve mapped the averaged intensity values to particle color and brightness for visualization. Neurons with low intensity were hidden to highlight active areas. By animating the hourly averaged data through frame interpolation, our visualization method intuitively revealed the spatial distribution and temporal dynamics of Ca^2+^ signals across the population of SCN neurons. The result is shown in Supplementary information, Video S1.

### Time predictor based on Ca^2+^ signals

Each dataset was divided into training, validation, and testing datasets at a ratio of 6:1:3. A CNN was constructed with one convolutional layer, a residual block of 32 channels followed by one FC layer. The residual block was implemented with two convolutional layers and a residual connection.^61^ Ca^2+^ signal across a predefined number of randomly selected neurons (e.g., 1, 10, 100, etc.) with the same time label was directly input to the CNN. Cross-entropy loss was used to control weight updates. Five random seeds, 0, 1, 2, 3 and 4 were used for these experiments, with 5000 trials each to assess time prediction accuracy. t-SNE analysis was performed on the output features of the second convolutional layer using random seed 0. All time predictors in this study shared the same configuration while each was trained with a whole-SCN dataset or a single-module dataset.

To quantify the contribution coefficient of each neuron in the SCN population to the time prediction, adjustments were made to both the datasets and the neural network architecture. First, the dataset was transformed to yield 24 signals obtained by Ca^2+^ signals from all neurons across the 24 time points. Then, the channels of the convolutional layers were reduced to 2, which was the minimal value to achieve 100% accuracy on the new dataset. Lastly, the integrated gradient-based attribution map^38^ was used to extract the contribution coefficients of individual neuronal Ca^2+^ signals to the time prediction. The contribution coefficients were normalized into the range of [0, 1] by min-max normalization.

### Classification of functional neuron subtypes via TraceContrast

The Ca^2+^ time series in consecutive 24 h (5-min recording in each hour) of all the observed neurons were represented as a 3D matrix. Each time series was randomly sampled into two overlapping sub-series and then input into an encoder. The encoder was composed of an FC layer, a timestamp masking module to mask latent vectors at randomly selected timestamps to generate an augmented context view, and a dilated CNN module with ten residual blocks.^62^ In particular, each sub-series was augmented by cropping and masking to generate similar sub-series (i.e., positive samples), and sub-series from different timestamps or various neurons were adopted as dissimilar sub-series (i.e., negative samples).^62^ In the cropping operation, a random length from each sub-sequence was selected and isolated. For the masking operation, we randomly generated mask values using a binomial distribution with a probability parameter of 0.5 (Python package function “numpy.random.binomial”). This implies an equal likelihood of a value being 0 or 1, where 1-mask retains the original data and 0-mask sets the corresponding data to zero. Further, to enhance positive sampling, each sub-series was augmented by introducing Gaussian noise to mimic noise generated during the acquisition process, and each sub-series was transformed into the wavelet domain with only the signal represented by high-frequency components retained as positive samples, based on the observation that the high-frequency signal captured Ca^2+^ bursts, the prominent feature for the functional neuron classification. The number of negative samples was the number of neurons multiplied by the length of the sequence for each time scale. Correspondingly, the number of positive samples was set to the number of negative samples, maintaining a 1:1 ratio for balanced training. The iteration process continued until the algorithm achieved convergence, typically occurring around 10 iterations. This augmentation process was uniformly applied to all sub-sequences, ensuring consistency across our dataset.

Hierarchical contrasting was then performed to learn invariant features at multiple resolutions of neurons and temporal sequences, and max pooling along the time axis was conducted to acquire the overall representation of each neuron. The network was optimized with temporal contrastive loss and instance-wise contrastive loss over multiple scales in a hierarchical framework.^62^ The K-means algorithm was finally deployed to cluster the overall neuronal representations into K categories.

### 3D visualization of the modular organization of functional neuron subtypes

The 3D positions of identified neurons were used to create a binary volume dataset, and each functional neuron subtype where the voxels in the volume surrounding the neurons were labeled as 1, while the remaining were labeled as 0. These volume datasets were further converted into a point cloud format and imported into MeshLab software (https://www.meshlab.net/). Within MeshLab, detailed triangle mesh was computed via Poisson surface reconstruction.^63^ Further manual repair and smoothing of the mesh was applied to improve mesh continuity. Neuron subtypes are represented in different colors. The result is shown in Supplementary information, Video S2.

### Quantification and statistical analysis

Data from each experiment represent at least three independent replicates. The number of independent experiments (*n*) and the relevant statistical parameters for each experiment (such as mean ± SEM) are described in the figure legends. Data fitting and calculation of the fitting coefficient (R^2^) were performed with MATLAB (Mathworks). Statistical significance was performed with MATLAB, and the *P* value was calculated by a two-sided Wilcoxon rank sum test, using MATLAB’s built-in function *ranksum*.

### Supplementary information

Supplementary information, Fig. S1

Supplementary information, Fig. S2

Supplementary information, Fig. S3

Supplementary information, Fig. S4

Supplementary information, Fig. S5

Supplementary information, Fig. S6

Supplementary information, Fig. S7

Supplementary information, Fig. S8

Supplementary information, Fig. S9

Supplementary information, Fig. S10

Supplementary information, Table S1

Supplementary information, Video S1

Supplementary information, Video S2

Supplementary Video Legend

## Data Availability

Raw data in this study and custom code used in this work are available at GitHub: https://github.com/RafaWong/SCN-Research-Project.
