# Supplementary material for: System-level time computation and representation in the suprachiasmatic nucleus revealed by large-scale calcium imaging and machine learning
Source: Cell Res. 2024 Apr 11;34(7):493–503. doi: 10.1038/s41422-024-00956-x (PMC11217450; doi:10.1038/s41422-024-00956-x)
Supplement: Supplementary file 1 — Supplementary information, Fig. S1 [file 41422_2024_956_MOESM1_ESM.pdf]

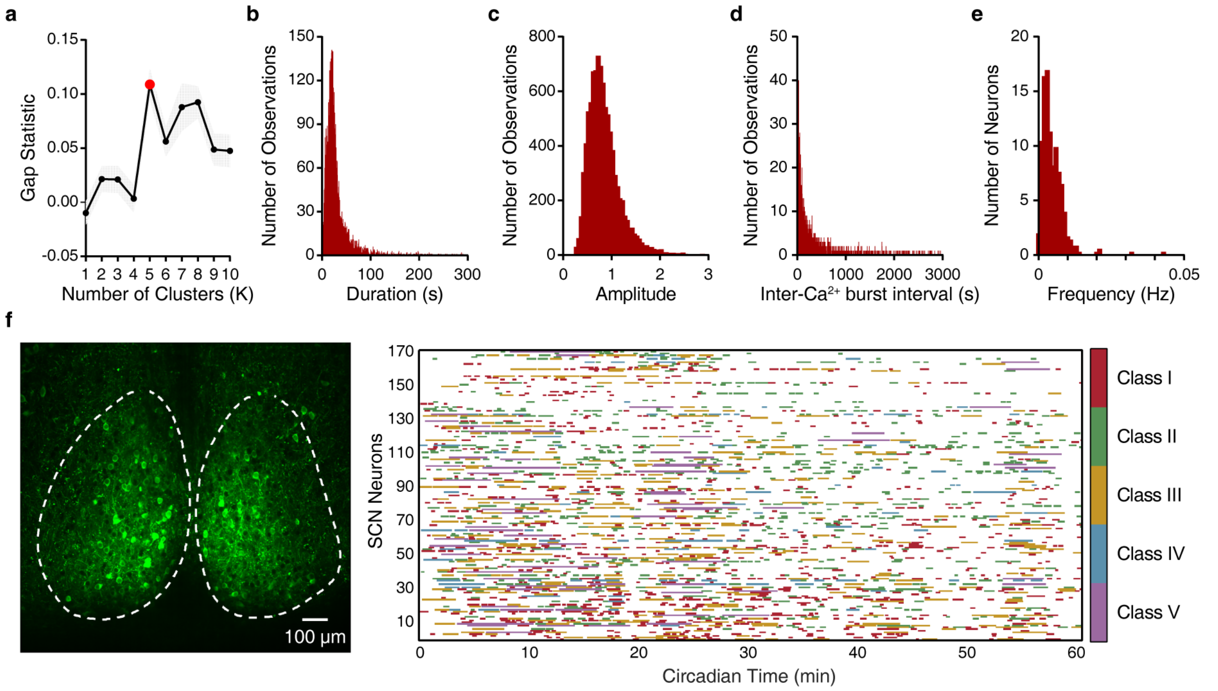

Fig. S1 Histogram distributions of Ca^{2+} burst attributes. a, Determining the number of Ca^{2+} burst clusters based on the Gap Statistic method. The gap statistic reaches its maximum at K = 5. **b**, Duration of Ca^{2+} bursts. 15 observations with durations greater than 300 s are omitted from the plot. **c**, Amplitude of Ca^{2+} bursts. 54 observations with amplitude greater than 3 $\Delta F/F$ are omitted from the plot. **d**, Intervals between consecutive Ca^{2+} bursts. 79 observations with intervals greater than 3 000 s are omitted from the plot. (n = 9 295 events from 354 neurons). **e**, Frequency of Ca^{2+} bursts in individual neurons. **f,** The same as Fig. 1a and 1f, except that data were from another SCN slice.
