# Supplementary material for: System-level time computation and representation in the suprachiasmatic nucleus revealed by large-scale calcium imaging and machine learning
Source: Cell Res. 2024 Apr 11;34(7):493–503. doi: 10.1038/s41422-024-00956-x (PMC11217450; doi:10.1038/s41422-024-00956-x)
Supplement: Supplementary file 2 — Supplementary information, Fig. S2 [file 41422_2024_956_MOESM2_ESM.pdf]

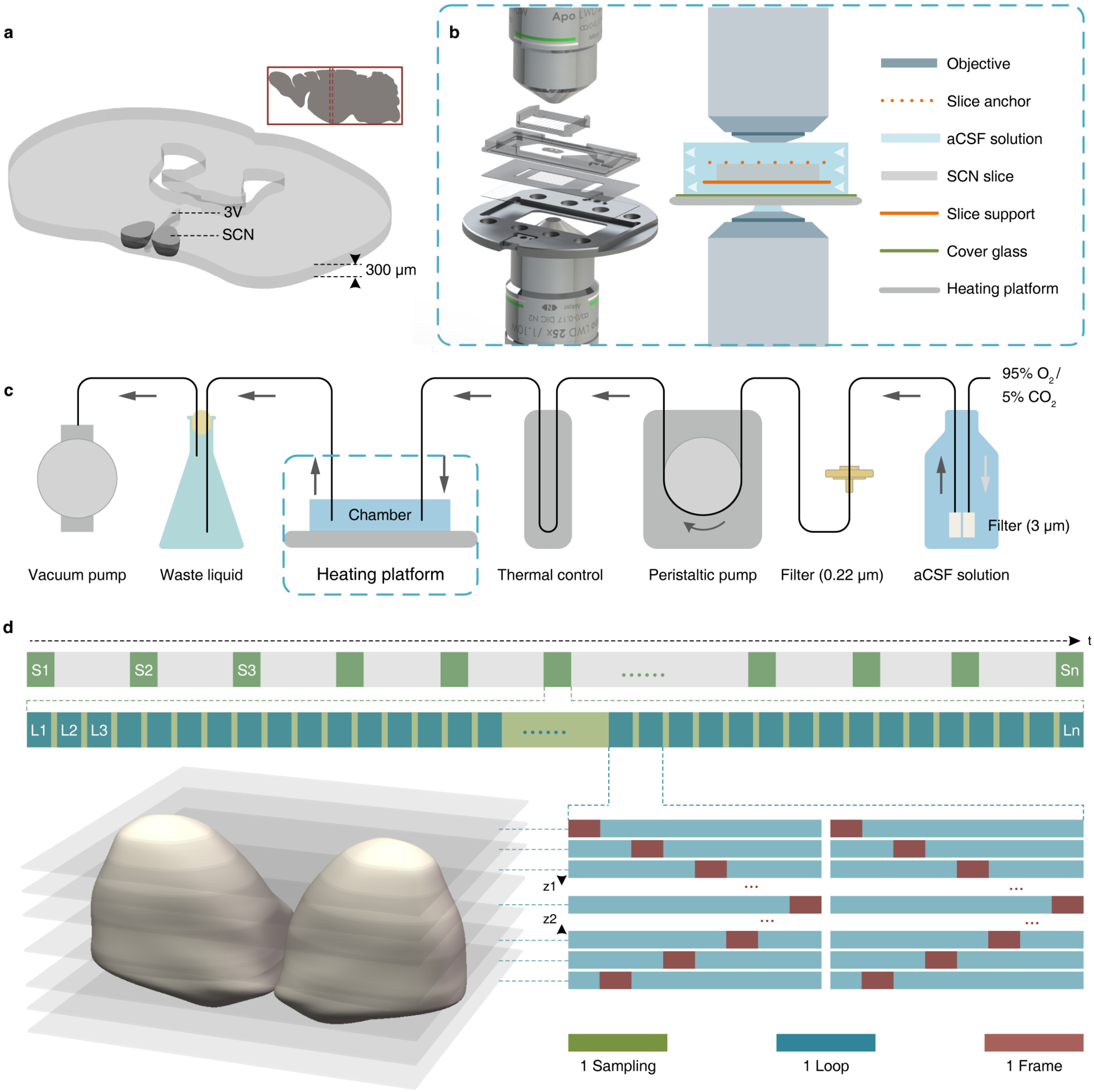

Fig. S2 Schematics of time-lapse volumetric Ca^{2+} imaging in adult SCN slice using dual-view two-photon microscopy. a, Cartoon illustration of a 300-μm coronal SCN slice. 3V: third ventricle. **b**, Imaging setup. Imaging used a dual-view (both upright and inverted) two-photon microscope equipped with a fast resonant scanner. The SCN slice is placed in a submerged slice chamber, which consists of a slice anchor, a slice support, a cover glass, and a cavity filled with aCSF. The submerged slice chamber is placed in a heating platform. **c**, The perfusion system including a gas supplement (95% O_2/5% CO_2), solution filtration (3-μm and 0.22-μm pore size filters), heating device (thermal control and a heating platform) and solution delivery

device (a peristaltic pump and a vacuum pump). **d**, Image acquisition protocol. For a 30-hour experimental session, three-dimensional image stacks were sampled every hour (S1, S2...S30) with a 5-min continuous recording time (L1, L2…L200). For a single loop (1 Loop), layers in the upper and lower z-stacks (z1, z2), each comprising 22–24 frames (1 frame per layer), were acquired alternately, at z-steps of ~6.7 μm and a volumetric imaging rate of ~0.67 volumes per second.
