# Supplementary material for: System-level time computation and representation in the suprachiasmatic nucleus revealed by large-scale calcium imaging and machine learning
Source: Cell Res. 2024 Apr 11;34(7):493–503. doi: 10.1038/s41422-024-00956-x (PMC11217450; doi:10.1038/s41422-024-00956-x)
Supplement: Supplementary file 3 — Supplementary information, Fig. S3 [file 41422_2024_956_MOESM3_ESM.pdf]

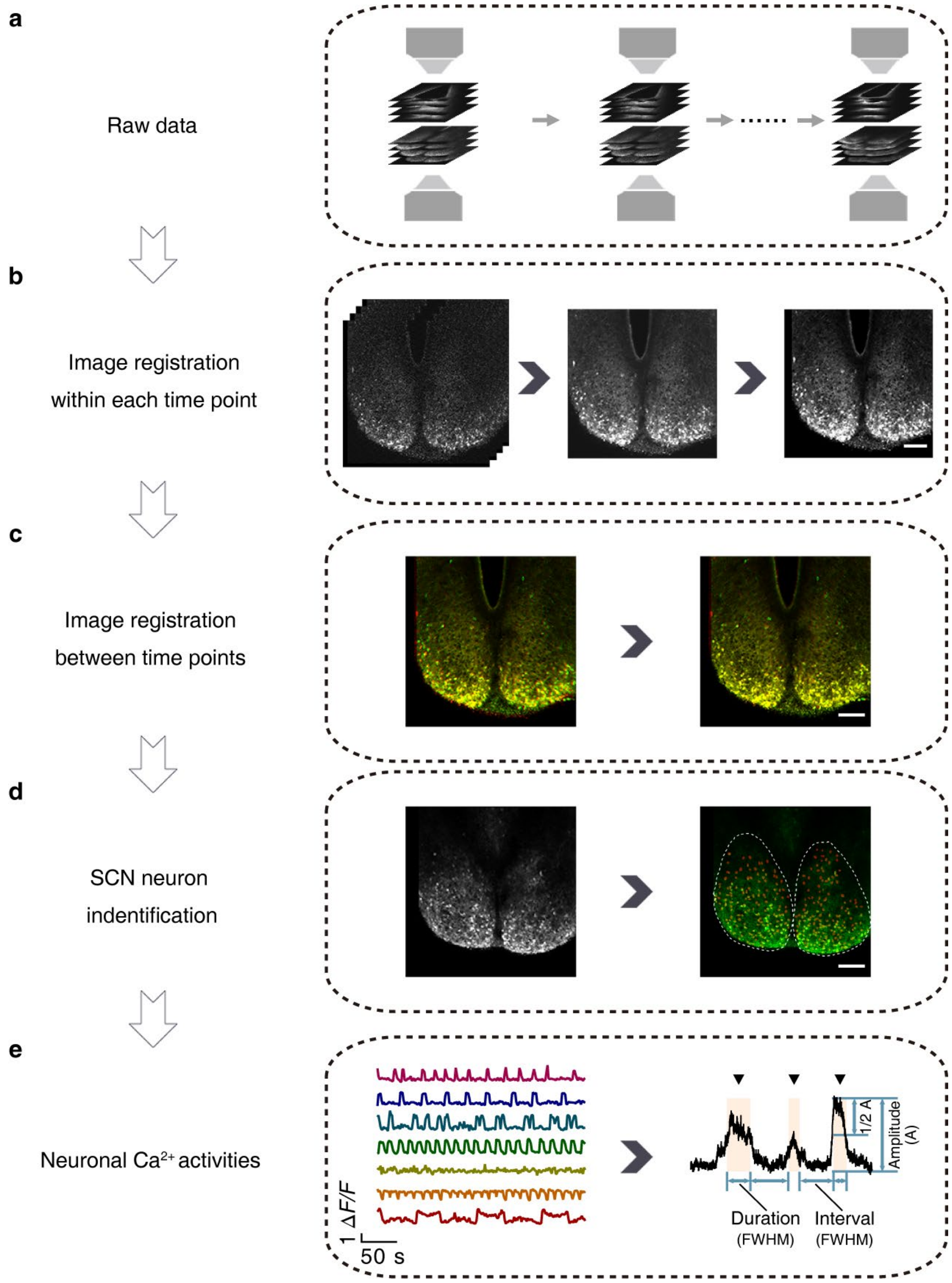

Fig. S3 Pipeline for automated image processing, neuronal segmentation, and parameter measurement based on 3D-t image stacks of SCN slices. a, Fusion and stitching of raw data of dual-view image stacks. **b**, Average-projection images after image registration within each time point. **c**, Image registration between time points. **d**, SCN neuron identification. Dashed lines show manually delineated SCN outer boundary. **e**, Extracted neuronal Ca^{2+} time series and parameter measurement. FWHM: full width at half maximum. Scale bar, 100 μm.
