# Supplementary material for: System-level time computation and representation in the suprachiasmatic nucleus revealed by large-scale calcium imaging and machine learning
Source: Cell Res. 2024 Apr 11;34(7):493–503. doi: 10.1038/s41422-024-00956-x (PMC11217450; doi:10.1038/s41422-024-00956-x)
Supplement: Supplementary file 4 — Supplementary information, Fig. S4 [file 41422_2024_956_MOESM4_ESM.pdf]

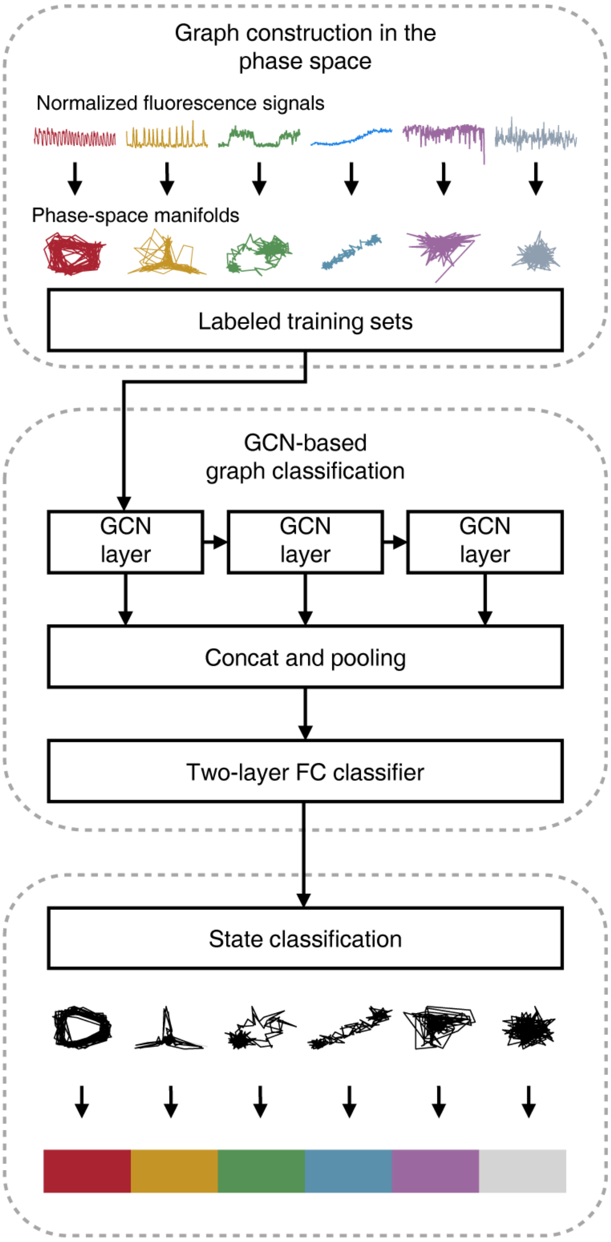

Fig. S4 Neuronal Ca^{2+} state classifier. Phase-space manifolds of 5-min z-score normalized fluorescence signals were used as the input. The classifier consists of three stacked graph convolutional (GCN) layers and two fully connected (FC) layers. The output of each graph convolutional layer is concatenated together. Then, a pooling operation is performed on the concatenated output. Finally, the pooled representation is fed into the following FC layers to obtain the state classification results. The classifier was trained with labeled datasets in a supervised manner.
