# Supplementary material for: System-level time computation and representation in the suprachiasmatic nucleus revealed by large-scale calcium imaging and machine learning
Source: Cell Res. 2024 Apr 11;34(7):493–503. doi: 10.1038/s41422-024-00956-x (PMC11217450; doi:10.1038/s41422-024-00956-x)
Supplement: Supplementary file 5 — Supplementary information, Fig. S5 [file 41422_2024_956_MOESM5_ESM.pdf]

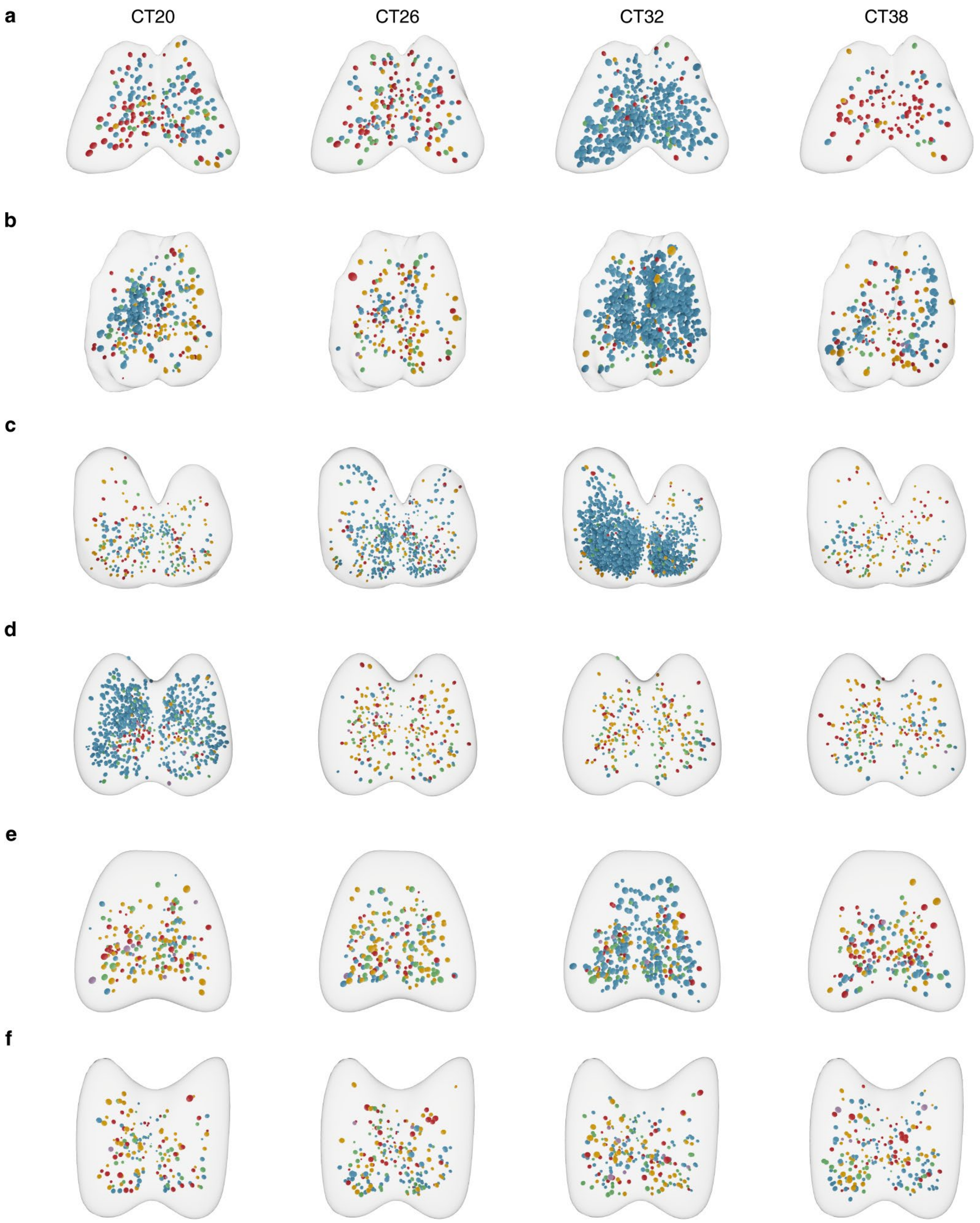

Fig. S5 Spatiotemporal pattern of neuronal Ca^{2+} states in SCN slices at 6-h intervals. The most populous State VI neurons are omitted for clarity. Note the dynamic changes of the mosaic patterns at different time of the day. The color code of Ca^{2+} states is the same as in Fig. 2.
