# Supplementary material for: System-level time computation and representation in the suprachiasmatic nucleus revealed by large-scale calcium imaging and machine learning
Source: Cell Res. 2024 Apr 11;34(7):493–503. doi: 10.1038/s41422-024-00956-x (PMC11217450; doi:10.1038/s41422-024-00956-x)
Supplement: Supplementary file 6 — Supplementary information, Fig. S6 [file 41422_2024_956_MOESM6_ESM.pdf]

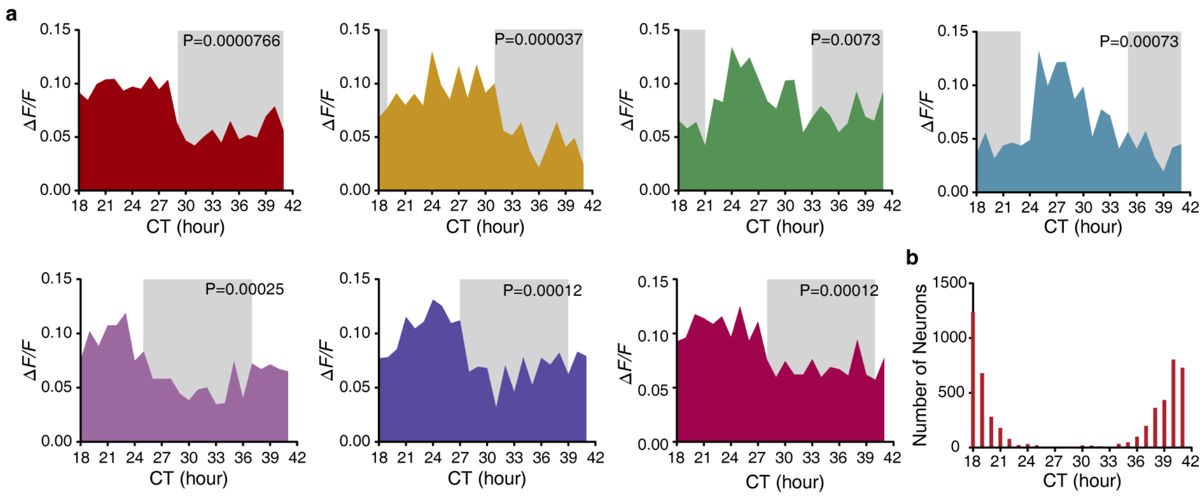

Fig. S6 Modes of single-neuron Ca^{2+} activities. Data were from a representative SCN slice containing 6 049 neurons. a, Representative single-neuron circadian rhythms in terms of Ca^{2+} signal amplitude. White and shadowed areas show two 12-hour divisions, corresponding to the high-activity Ca^{2+} mode (H-mode) and low-activity Ca^{2+} mode (L-mode) of individual neurons, respectively. Note the distinctive phases and time courses among different neurons. *P* value is determined by a two-sided Wilcoxon rank sum test. **b**, Timing of mode switching among SCN neurons. In this analysis, 5 326 neurons (88%) displayed circadian rhythmicity. Timing is determined as the onset of the H-mode for the 12-to-12-hour sliding window when the H- to L-mode difference is most significant.
