# Supplementary material for: System-level time computation and representation in the suprachiasmatic nucleus revealed by large-scale calcium imaging and machine learning
Source: Cell Res. 2024 Apr 11;34(7):493–503. doi: 10.1038/s41422-024-00956-x (PMC11217450; doi:10.1038/s41422-024-00956-x)
Supplement: Supplementary file 7 — Supplementary information, Fig. S7 [file 41422_2024_956_MOESM7_ESM.pdf]

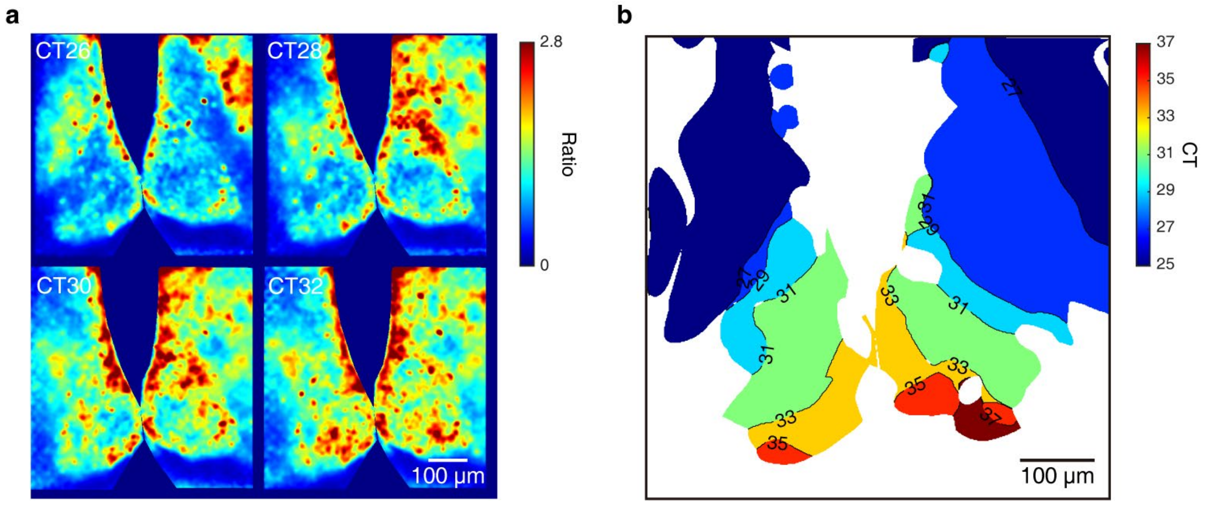

Fig. S7 Visualization of PWHA traversing the entire SCN. a, Heatmap of PWHA in a representative layer of a 300-μm SCN slice at CT26, CT28, CT30, and CT32. Scale bar, 100 μm. **b**, Contour plot of PWHA in this layer, with CTs corresponding to different contours denoted by color. Scale bar, 100 μm.
