# Supplementary material for: System-level time computation and representation in the suprachiasmatic nucleus revealed by large-scale calcium imaging and machine learning
Source: Cell Res. 2024 Apr 11;34(7):493–503. doi: 10.1038/s41422-024-00956-x (PMC11217450; doi:10.1038/s41422-024-00956-x)
Supplement: Supplementary file 8 — Supplementary information, Fig. S8 [file 41422_2024_956_MOESM8_ESM.pdf]

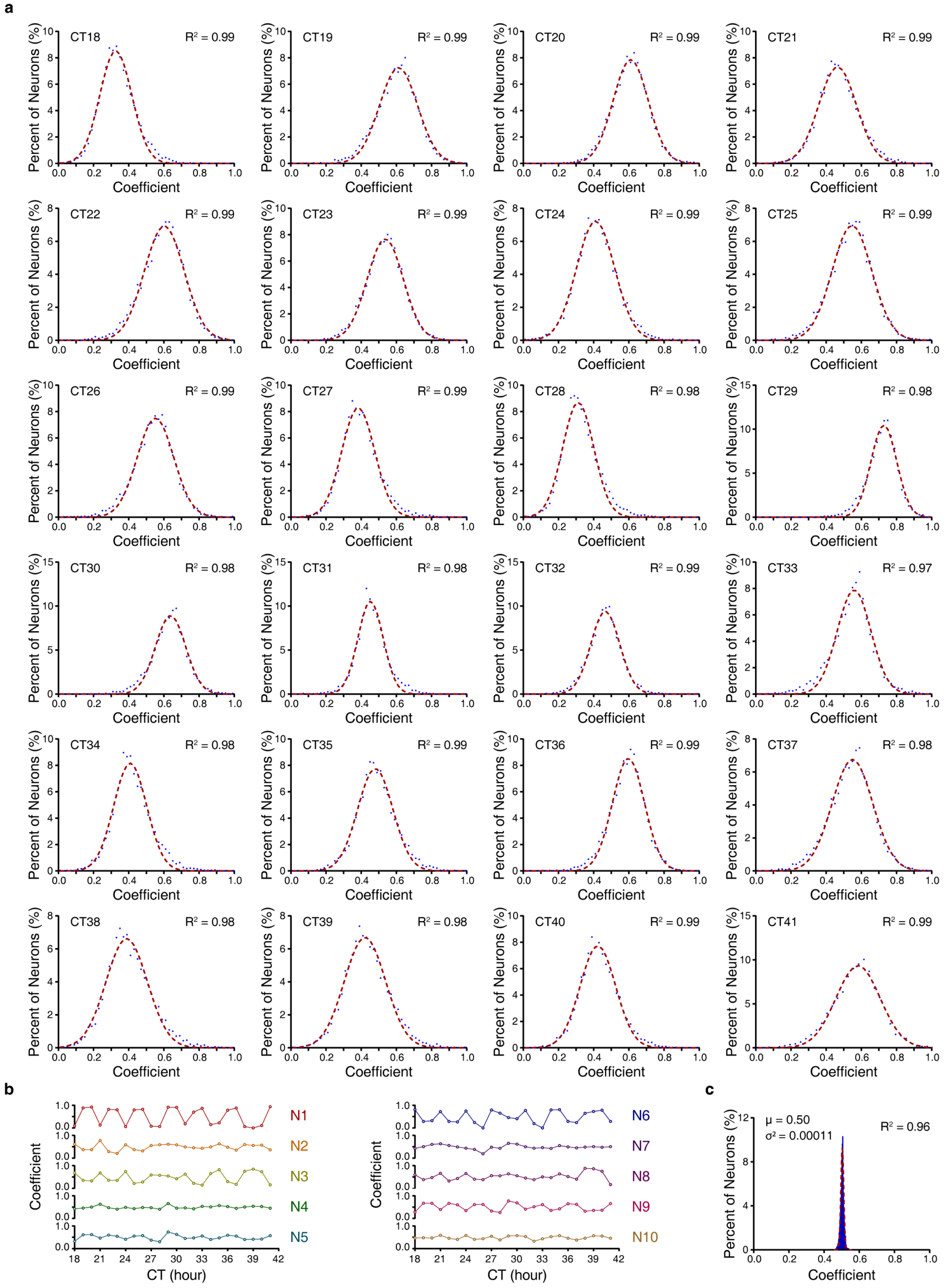

Fig. S8 Contribution of individual SCN neurons to hourly time prediction. a, Histograms of contribution coefficients of neurons at different time points. Red dashed line in each panel represents the best-fitted Gaussian function, and R^2 refers to the fitting coefficient. Dataset was

the same as in Fig. 3e. **b**, Representative time courses of neuronal contribution coefficients, showing fluctuations over an intact circadian period. **c**, Histogram of 24-hour averaged contribution coefficients of individual SCN neurons. Top left: values of the mean (μ) and variance (σ^2) of the best-fitted Gaussian function.
