# Supplementary material for: System-level time computation and representation in the suprachiasmatic nucleus revealed by large-scale calcium imaging and machine learning
Source: Cell Res. 2024 Apr 11;34(7):493–503. doi: 10.1038/s41422-024-00956-x (PMC11217450; doi:10.1038/s41422-024-00956-x)
Supplement: Supplementary file 9 — Supplementary information, Fig. S9 [file 41422_2024_956_MOESM9_ESM.pdf]

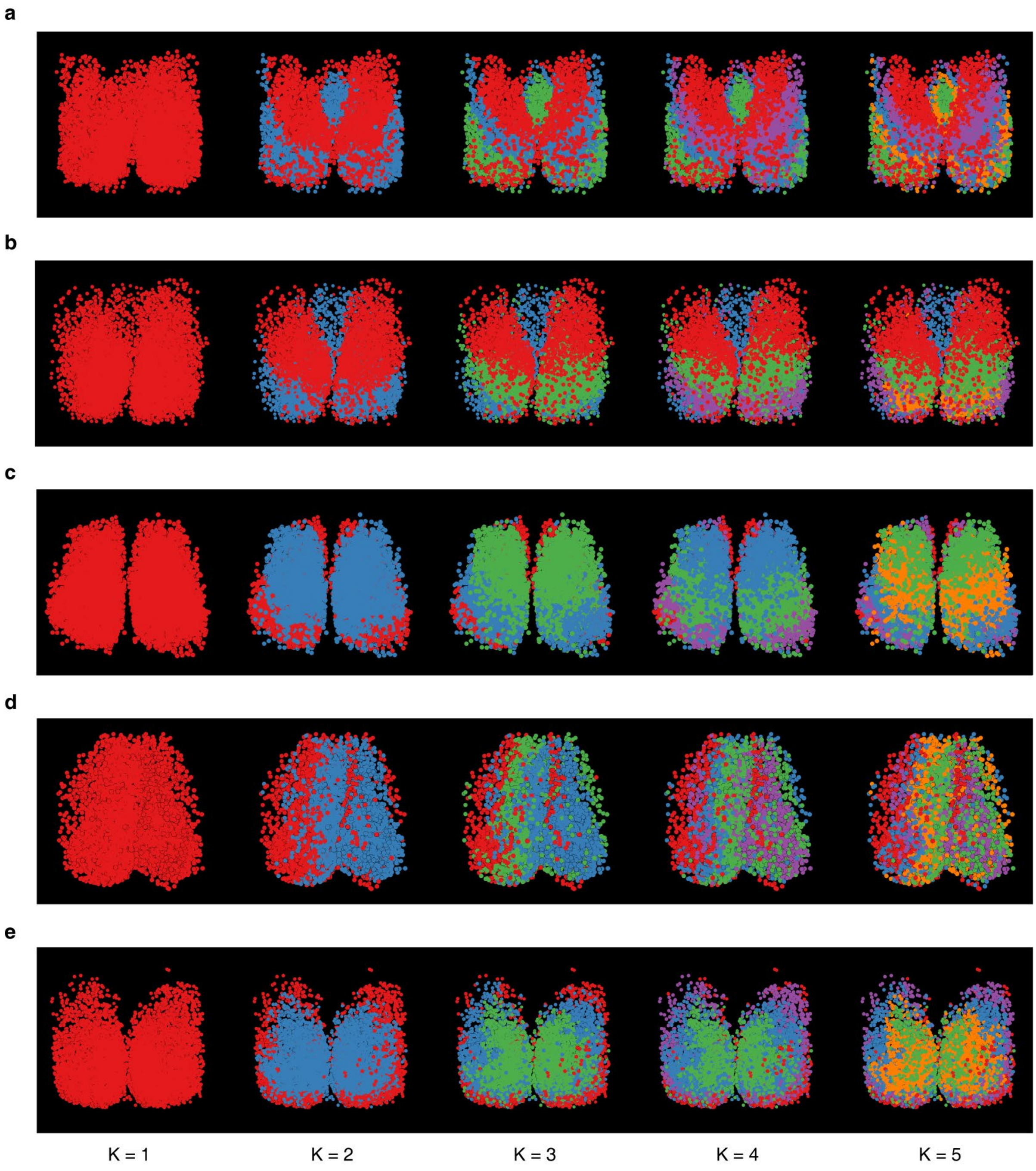

Fig. S9 Related to Fig. 4b: additional examples of neuron subtype classification via TraceContrast. The predefined number of clusters (K) is listed at the bottom.
