# Supplementary material for: System-level time computation and representation in the suprachiasmatic nucleus revealed by large-scale calcium imaging and machine learning
Source: Cell Res. 2024 Apr 11;34(7):493–503. doi: 10.1038/s41422-024-00956-x (PMC11217450; doi:10.1038/s41422-024-00956-x)
Supplement: Supplementary file 10 — Supplementary information, Fig. S10 [file 41422_2024_956_MOESM10_ESM.pdf]

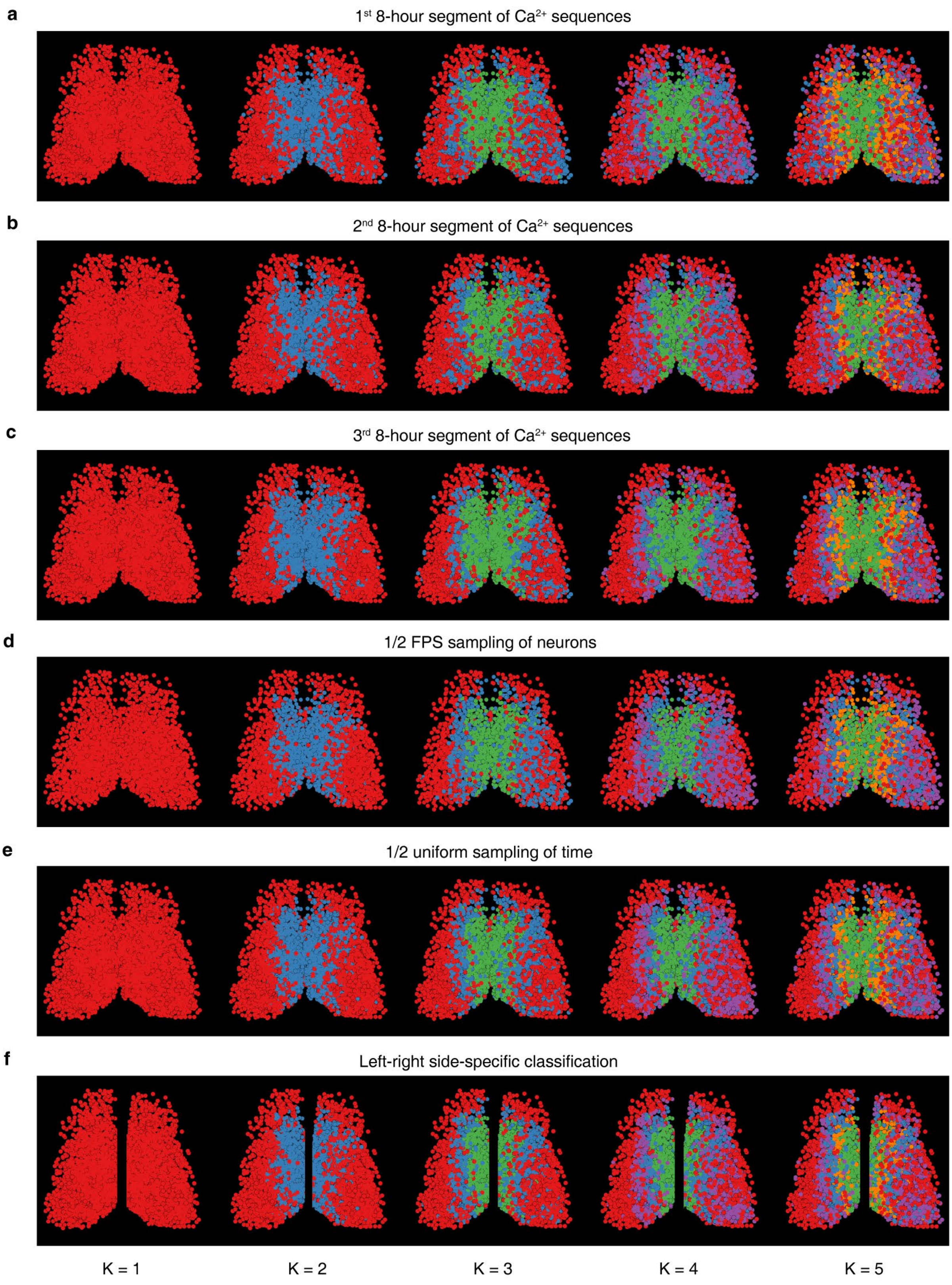

Fig. S10 Robustness and stability of modular time feature representation as reported in Fig. 4. Dataset was the same as in Fig. 4. **a–c**, Neuron subtype classification results based on the first (**a**, CT18–CT25), second (**b**, CT26–CT33), and third (**c**, CT34–CT41) segment of

Ca^{2+} sequences in consecutive 24 hours. Note that in the first segment, there was no PWHA but the hierarchical modularity remained intact, indicating that such an emergent pattern is not dependent on PWHA. **d–f**, Neuron classification results under 1/2 farthest point sampling (FPS) of neurons spatially (**d**), 1/2 uniform sampling of Ca^{2+} recordings temporally (**e**) and single-sided analysis using either left or right SCN data independently (**f**). The predefined number of clusters (K) is listed at the bottom.
