# Supplementary material for: System-level time computation and representation in the suprachiasmatic nucleus revealed by large-scale calcium imaging and machine learning
Source: Cell Res. 2024 Apr 11;34(7):493–503. doi: 10.1038/s41422-024-00956-x (PMC11217450; doi:10.1038/s41422-024-00956-x)
Supplement: Supplementary file 11 — Supplementary information, Table S1 [file 41422_2024_956_MOESM11_ESM.pdf]

**Table S1 General information of six datasets**

| Datasets | I | II | III | IV | V | VI |
|---|---|---|---|---|---|---|
| Animal model | *Viaat-Cre::GCaMP6s* male mice (6–8 weeks) | | | | | |
| Time slot | 5-min recording per hour, from CT12 to CT41 | | | | | |
| Sampling rate | 0.67 vps | 0.61 vps | 0.61 vps | 0.62 vps | 0.62 vps | 0.61 vps |
| Neurons | 6 049 | 6 445 | 8 968 | 8 229 | 7 782 | 7 828 |

*vps: volumes per second, for volumetric imaging rate
