# Supplementary material for: System-level time computation and representation in the suprachiasmatic nucleus revealed by large-scale calcium imaging and machine learning
Source: Cell Res. 2024 Apr 11;34(7):493–503. doi: 10.1038/s41422-024-00956-x (PMC11217450; doi:10.1038/s41422-024-00956-x)
Supplement: Supplementary file 14 — Supplementary Video Legend [file 41422_2024_956_MOESM14_ESM.pdf]

Video S1 4D visualization of PWHA traversing the entire SCN. Related to Supplementary information, Fig. S7.

Video S2 3D visualization of the modular organization of functional neuron subtypes. Related to Fig. 4b with K = 5.
